# Improving Estimations of Spatial Distribution of Soil Respiration Using the Bayesian Maximum Entropy Algorithm and Soil Temperature as Auxiliary Data

**DOI:** 10.1371/journal.pone.0146589

**Published:** 2016-01-25

**Authors:** Junguo Hu, Jian Zhou, Guomo Zhou, Yiqi Luo, Xiaojun Xu, Pingheng Li, Junyi Liang

**Affiliations:** 1 Information Engineering College of Zhejiang A & F University, Linan, PR China; 2 Zhejiang Provincial Key Laboratory of Forestry Intelligent Monitoring and Information Technology Research, Linan, PR China; 3 Department of Microbiology and Plant Biology, University of Oklahoma, Norman, Oklahoma, United States of America; University of Connecticut, UNITED STATES

## Abstract

Soil respiration inherently shows strong spatial variability. It is difficult to obtain an accurate characterization of soil respiration with an insufficient number of monitoring points. However, it is expensive and cumbersome to deploy many sensors. To solve this problem, we proposed employing the Bayesian Maximum Entropy (BME) algorithm, using soil temperature as auxiliary information, to study the spatial distribution of soil respiration. The BME algorithm used the soft data (auxiliary information) effectively to improve the estimation accuracy of the spatiotemporal distribution of soil respiration. Based on the functional relationship between soil temperature and soil respiration, the BME algorithm satisfactorily integrated soil temperature data into said spatial distribution. As a means of comparison, we also applied the Ordinary Kriging (OK) and Co-Kriging (Co-OK) methods. The results indicated that the root mean squared errors (RMSEs) and absolute values of bias for both Day 1 and Day 2 were the lowest for the BME method, thus demonstrating its higher estimation accuracy. Further, we compared the performance of the BME algorithm coupled with auxiliary information, namely soil temperature data, and the OK method without auxiliary information in the same study area for 9, 21, and 37 sampled points. The results showed that the RMSEs for the BME algorithm (0.972 and 1.193) were less than those for the OK method (1.146 and 1.539) when the number of sampled points was 9 and 37, respectively. This indicates that the former method using auxiliary information could reduce the required number of sampling points for studying spatial distribution of soil respiration. Thus, the BME algorithm, coupled with soil temperature data, can not only improve the accuracy of soil respiration spatial interpolation but can also reduce the number of sampling points.

## Introduction

Soil respiration represents one of the most important fluxes in the terrestrial carbon (C) cycle [1–3]. Therefore, accurately estimating the amount of soil carbon dioxide (CO_2_) efflux is of great importance to understand the terrestrial C cycle and the mechanisms involving climate change and its effects. Influenced by numerous natural factors, soil CO_2_ efflux tends to show intense spatial heterogeneity [4, 5]. Practically, studies on soil CO_2_ efflux generally adopt the scattered point sampling method because of the measurement limits associated with soil respiration[5, 6]. However, due to the extreme spatial and temporal variabilities in soil respiration, it is crucial to have denser spatial data points to undertake spatial interpolation [7].

There are various spatial interpolation methods, most of which have already been applied in many fields [8]. Generally, spatial interpolation methods can be classified into non-geostatistical methods, geostatistical methods (e.g., Kriging), and mixed methods. As an unbiased estimate method, the Kriging method is the most mature and popular method in the field of environmental science[9–11]. Due to expanding application scopes and varying application requirements in different fields, mixed interpolation methods have developed over time, both in theory and application [12]. In addition to a number of commonly used methods, some machine-learning methods have also been adopted toward spatial interpolation, and fairly good results were derived. Popular machine-learning methods include the neural network method and random forest method[13, 14]. Li et al. [15] explored the utilization of many machine-learning methods in spatial interpolation. Hu et al. [16] employed the neural network interpolation algorithm to study illumination distributions. Furthermore, many other algorithms are already being used to study interpolation in the field of environmental science. For instance, researchers have expanded data use to spatial and temporal aspects by adopting Bayesian prior information [17, 18]. Most of the current interpolation methods have already been applied in the study of spatial distribution of soil respiration. Teixeira et al. [19] compared the results of the application of the Kriging and sequential Gaussian fitting methods to soil respiration interpolation, and they found that latter achieved better results. Stoyan et al. [20] studied the spatial variation of soil respiration using the Kriging method. Jordan et al. [21] also used the Kriging method to study the small-scale spatial heterogeneity of soil respiration for a growing forest. However, soil respiration entails a complex a complex interrelationship of physical, biological, and chemical reactions, and thus, it is hard to fully analyze its spatial heterogeneity by merely interpolating data from a few sampled points. Consequently, it is crucial to improve the interpolation accuracy of soil respiration and compensating for the above-mentioned deficiency by including additional information, such as impact factors that are easily accessible, for example, auxiliary information on soil respiration, during sampling. Teixeira et al. [6] compared the interpolation results obtained from the OK and Co-Kriging (Co-OK) methods, using soil bulk density as the second feature, and they showed that the inclusion of this feature greatly improved the effect of interpolation. Huang et al. [22] studied the influence of vegetation and soil properties on the estimation of soil respiration space with the aid of remote sensing techniques. Jurasinski et al. [23] took root microbes as auxiliary information and investigated the spatial distribution of soil respiration by adopting the Co-OK method.

The Bayesian Maximum Entropy (BME) algorithm is a combination of the Bayesian statistical theory and the information theory of Shannon. It is used to handle spatiotemporal variables that can be integrated into more empirical knowledge and soft data (auxiliary information), and consequently, it can aid in the collation of environmental information in the field of geostatistics. Compared to the kriging algorithm, the BME algorithm is more theoretical and systematic. Gao et al. [24] coupled the temperature data obtained by remote sensing as soft data with the BME algorithm to study the regional spatial distribution of soil moisture. They compared the results of the BME algorithm to those of kriging and proved that the former could improve the regional interpolation effect. Akita et al. [25] developed a moving-window BME method to improve the estimation accuracy of regional air pollutant distribution.

Studies in the field of soil respiration have indicated that soil surface temperature significantly affects soil respiration, representing an exponential relationship [26–28]. Soil temperature can be more easily obtained by advanced technologies (such as wireless sensor networks) than soil respiration. Thus, in this paper, we take advantage of the ability of the BME algorithm to use soft data and employ soil temperature as the soft data. In doing so, we confirm the following three hypotheses: (1) The BME method is more accurate at estimating the spatial distribution of soil CO_2_ efflux than OK and Co-OK methods, (2) data on soil temperature, used as auxiliary information, provide improved estimates of the spatial distribution of soil CO_2_ efflux on small scale, (3) and this auxiliary information can help reduce the number of sampling points while studying the spatial distribution of soil CO_2_ efflux.

## Materials and Methods

### Study area

The experimental site was located in the city of Lin’an in the northwest of Jincheng County, Zhejiang Province, China (119°43’15.24”–119°43’26.97”E and 30°15’21.60”–30°15’33.27”N). The entire study area is open grassland bounded by a lake in the east; sparse woods in the south, west, and northwest; and open grassland in the northeast. The average altitude is 50 m, and the highest altitude is 170 m. The area has an average annual frost-free period of 237 days and receives an average annual rainfall of 1613.9 mm over a total of about 158 days. The average annual temperature is 16.4°C, with 1847.3 h of annual sunshine. Roughly speaking, this area is warm and humid, featuring a subtropical monsoon climate, and it has sufficient illumination, abundant rainfall, and four distinct seasons.

The area belongs to Zhejiang A & F University, and we can do research freely. Other researchers also can easily get permission to do research in the area. Warning signs had been set during the testing process to insure there was no any danger. The research didn’t cause irreversible damage to the soil and there isn’t any protected species in the study area.

### Data sources

The experimental area covers an area of 35 m × 35 m, which was divided into grids of 5 × 5m. Seven Lr100GE-6400 [29], developed by GreenOrbs Laboratory, were used to measure the CO_2_ efflux within each grid. The Lr100GE-6400 is an open-box CO_2_ efflux measuring instrument. One day before measuring the CO_2_ efflux, a PVC soil collar was pressed into the soil to a depth of about 5 cm at the center of the surface of each soil core. In order to ensure simultaneous readings, we took 1 minute to warm up the 7 instruments, and 3 minutes to conduct the measurements, with all measurements being completed within the 90 minutes between 13:00 to 14:30 on September 30 (Day 1) and October 7 (Day 2), 2014. We chose the stable data as the experimental data from all the observed data. On Day 1, we conducted measurements from east to west (horizontal direction), while on Day 2, we did so from south to north (vertical direction). The summary statistics of the CO_2_ efflux data have been provided in Table 1. Higher density temperature data (35 × 35) were measured manually by 30 corrected soil TP-101 thermometers, logged when they achieved equilibrium at 1 to 2 min inserted into the soil at a depth of about 5 cm-10cm. All rounds of sampling were completed in 90 min. The summary statistics of the soil temperature data appear in Table 2.

**Table 1:** Summary of Soil Respiration Data.

ID	Date	No.	Max(μmg/m^2^s)	Min(μmg/m^2^s)	Range(μmg/m^2^s)	Mean(μmg/m^2^s)	SD(μmg/m^2^s)	Cv(%)
**Day 1**	September 30, 2014	49	4.939	2.242	2.698	3.476	0.602	17.332
**Day 2**	October 7, 2014	49	6.649	3.635	3.019	5.810	0.512	8.805

**Table 2:** Summary of Soil Temperature Data.

ID	Date	No.	Max (°C)	Min (°C)	Range (°C)	Mean (°C)	SD (°C)	Cv (%)
**Day 1**	September 30, 2014	1225	26.58	25.16	1.42	25.89	0.36	1.371
**Day 2**	October 7, 2014	1225	27.70	25.98	1.72	27.18	0.4	1.468

### Data preprocessing

The application of soft data (soil temperature) is important for the BME algorithm, to integrate uncertain information into the estimation. Suitable and high quality soft data can improve the performance of the algorithm. Soft data can be integrated into expert knowledge, experimental conclusions, and so on, with the common probability-type of soft data [30, 31]. Probability soft data can be approximately normally distributed or Student *t*-distributed to express the measurement error or physical interpretation [32, 33]. Interval soft data denote physical meanings with upper and lower bounds. After reviewing other similar studies, we assumed that soil respiration and soil temperature share a functional relationship of the Arrhenius type [26, 27], as shown in Eq 1. We used the measured data to calculate the fitting parameters in Eq 1. The soft data are given by the fitting results at regular intervals of the Student’s *t*-distribution, and the formula used to calculate the prediction interval is given by Eq 2 [34]:

$$\hat{R_{s}}=ae^{b*T}$$ (1)

$$R_{s\cdot interval}\;=\;{\hat{R}}_{s\cdot i}\pm t_{n-2,0.025}S_{T\cdot P}\sqrt{1+\frac{1}{n}+\frac{{\left(T_{i}-\bar{T}\right)}^{2}}{S_{TT}}}$$ (2)

**Fig 1:**
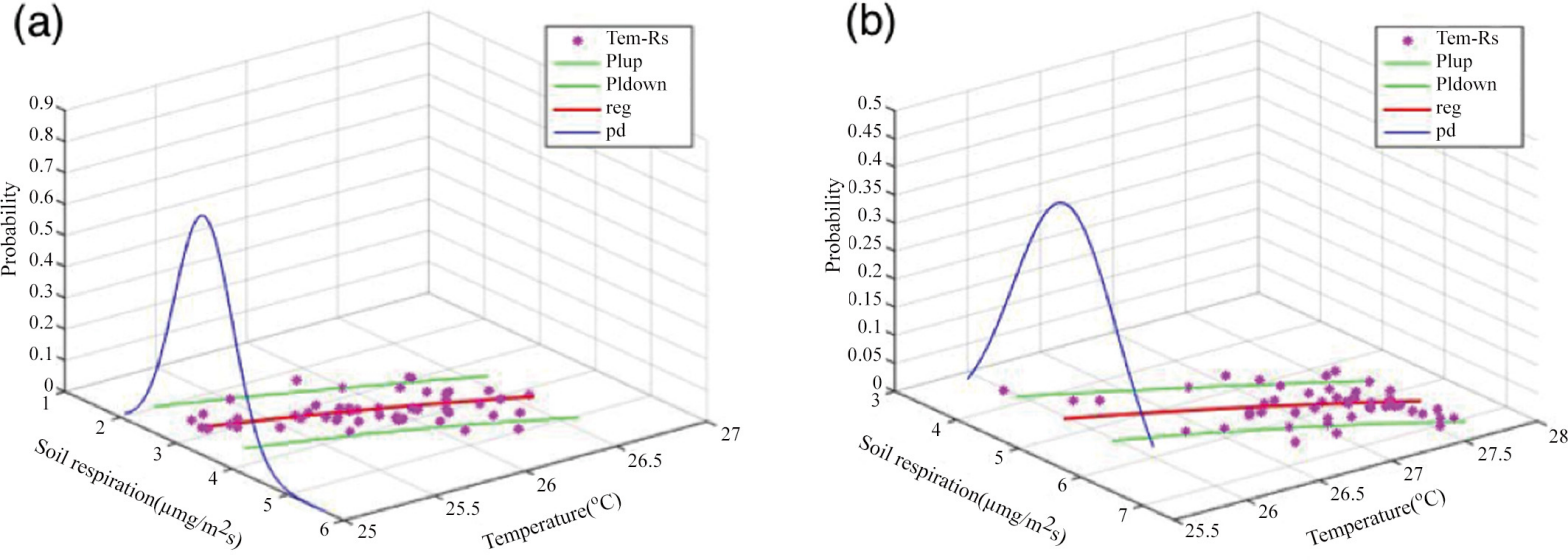
Temperature–soil CO_2_ Efflux Scatter Plots (*Tem*–*R*_*s*_), Fitting Relationship, and Probability Distribution. Plots (a) and (b) denote the aforementioned relationships for Day 1 and Day 2, respectively. The red line (*reg* in the legend) shows the relationship between temperature and soil CO_2_ efflux. The green lines (*Plup* and *Pldown* in the legend) indicate the prediction intervals at a confidence level of 95%. The blue line (*pd* in the legend) refers to the probability distribution of the soft data (estimated CO_2_ efflux corresponding to soil temperature).

Here, $\hat{R_{S}}$ is the estimated soil CO_2_ efflux relative to the soil temperature *T*_*i*_, with parameters *a* and *b*. *R*_*S*⋅*interval*_ is the prediction interval corresponding to each estimated soil CO_2_ efflux ${\hat{R}}_{s\cdot i}.\;t_{n-2,0.025}$ is the critical value of the Student’s *t*-distribution with (*n*– 2) degrees of freedom and a confidence level of 95%. *S*_*T*⋅*P*_ is the standard deviation (SD) of the soil CO_2_ efflux estimation error. $\bar{T}$ is the average soil temperature. *S*_*TT*_ is the sum of the square of the deviations. The numerical values of parameters in Eqs 1 and 2 are shown in Table 3. Fig 1 shows the relationship between soil CO_2_ efflux and soil temperature, the probability distribution, and prediction interval of the soft data.

**Table 3:** Parameters of the Arrhenius Type Formula and Summary Statistics of the Soft Data.

ID	CR	*a*	*b*	*S*_*T·P*_	*S*_*TT*_	$\bar{T}$	*t*_*n*–2,0.025_
**Day 1**	0.6058	0.0037	0.3497	0.65	6.38	25.89	1.761
**Day 2**	0.5701	0.1331	0.1370	0.74	8.28	27.18	1.761

Note: The parameters in this table were calculated considering the 49-point measuring scheme, and they would need to be recalculated if a different sample size was used.

### Comparison between methods

This paper compared the results of the BME method and two Kriging methods (OK and Co-OK). The spatial estimates from the three methods were evaluated by several validation methods.

#### Kriging

Kriging is an unbiased linear estimation method used to characterize a physical attribute's spatial variation and generate attribute estimates at un-sampled locations. OK, the simplest and most widely used kind of Kriging, calculates the weights (relative contributions) of attribute samples surrounding each estimation point by means of the geostatistical variogram, and the unknown attribute values are then estimated as the linear combination of the weighted samples, subject to the condition that the sum of the weights is equal to 1, see Eq 3:

$$Z*\left(V_{0}\right)\;=\;\sum_{i=1}^{n}\lambda_{i}Z\left(V_{i}\right),\;with\;\sum_{i=1}^{n}\;\lambda_{i}\;=\;1$$ (3)

Here, Z*(*V*_0_) represents the value of the estimated point *V*_0_, *V*_*i*_ represents the value of the *i-*th point among *n* points around *V*_0_, and *λ*_*i*_ denote the weight coefficients.

Co-OK follows the same principle as OK. However, the former considers more than one variable, and in addition to considering the spatial relationship of the main variable itself, it considers the relationships between the main variable and all other variable types to enable better predictions. Adding more information about relevant variables while estimating the main variable can improve the estimated effects. As we consider the spatial variability of soil C flux over time, adding the closely related variable of soil temperature can compensate for the insufficiency of CO_2_ efflux sampling and improve the accuracy of the estimation, as shown in Eq 4:

$$Z_{1}^{*}\left(V_{0}\right)\;=\;\sum_{i=1}^{n_{1}}\lambda_{1i}Z_{1}\left(V_{1i}\right)+\sum_{j=1}^{n_{2}}\lambda_{2j}Z_{2}\left(V_{2j}\right),\;with\;\sum_{i=1}^{n_{1}}\lambda_{1i}=1\;and\;\sum_{j=1}^{n_{2}}\lambda_{2j}\;=\;1$$ (4)

Here, $Z_{1}^{*}$ is the estimate of the main variable *Z*_1_ at point *V*_0_. *λ*_1*i*_ is the weight of the main variable *Z*_1_, and *λ*_2*j*_ is the weight of the auxiliary variable *Z*_2_ (the second characteristic).

The Kriging method studies the spatial relationships from point to point, which are usually used to express spatial variability with an experimental variogram. Variograms generally include self-variograms and cross-variograms, and they are used to determine the spatial autocorrelations of the variable’s properties, as shown as Eq 5 [35]:

$$\hat{\gamma }\left(h\right)=\frac{1}{2N\left(h\right)}\underset{i=1}{\sum^{N\left(h\right)}}{\left[Z\left(X_{i}\right)-Z\left(X_{i}+h\right)\right]}^{2}$$ (5)

where $\hat{\Upsilon }\left(h\right)$ is the experimental semivariance at a separation distance *h*, *Z*(*X*_*i*_) is the property value of the variable at the *i*-th point, and *N*(*h*) is the number of pairs of points separated by the distance *h*.

In this paper, we added soil temperature properties as the auxiliary information. Cross-variograms can show the relationship between two variables, as seen in Eq 6 [36]:

$${\hat{\gamma }}_{ZY}\left(h\right)=\frac{1}{2N\left(h\right)}\sum_{i=1}^{N\left(h\right)}\left[Z\left(X_{i}\right)-Z\left(X_{i}+h\right)\right]\left[Y\left(X_{i}\right)-Y\left(X_{i}+h\right)\right]$$ (6)

${\hat{\gamma }}_{ZY}\left(h\right)$ is the experience cross-variogram at separation distance *h*, *Z* (*X*_*i*_) is the main property value at the *i-*th point, *Y*(*X*_*i*_) is the secondary property value at the *i-*th point, and *N*(*h*) is the number of pairs of points separated by distance *h*.

Based on the coefficient of determination (*R*^2^) and squared residuals, we chose the Gaussian and Spherical models as the optimal variogram model, as depicted by Eq 7 and Eq 8:

$$\gamma (h)=c_{0}+c(1-exp(-3\frac{h^{2}}{a^{2}}))$$ (7)

$$\gamma (h)=c_{0}+c[\frac{3}{2}(\frac{h}{a})-\frac{1}{2}{\left(\frac{h}{a}\right)}^{3}]$$ (8)

where *γ* (*h*) is the semivariance, *C*_0_ indicates the nugget, *C* represents the structural variability, *C*_0_ + *C* represents the sill variance, and *a* denotes the correlation length range in geostatistics.

#### Bayesian Maximum Entropy

BME is a spatiotemporal analysis and mapping method that combines information theory with Bayesian statistics [30]. Compared with classical geostatistics (kriging), BME can consider general-prior and site-specific knowledge using a certain error and uncertainty in soft (uncertain) data, in addition to hard (exact) data, to improve the accuracy of spatiotemporal analysis. The meaning of soft data is very flexible; it may denote sampled data, historical data, rough measurement data, expert knowledge, and/or model fitting data. The manner in which the BME method uses comprehensive information and employs the probability method to express uncertainty to the extent possible allows it to present more realistic results in the spatiotemporal analysis of nature attributes.

**Fig 2:**
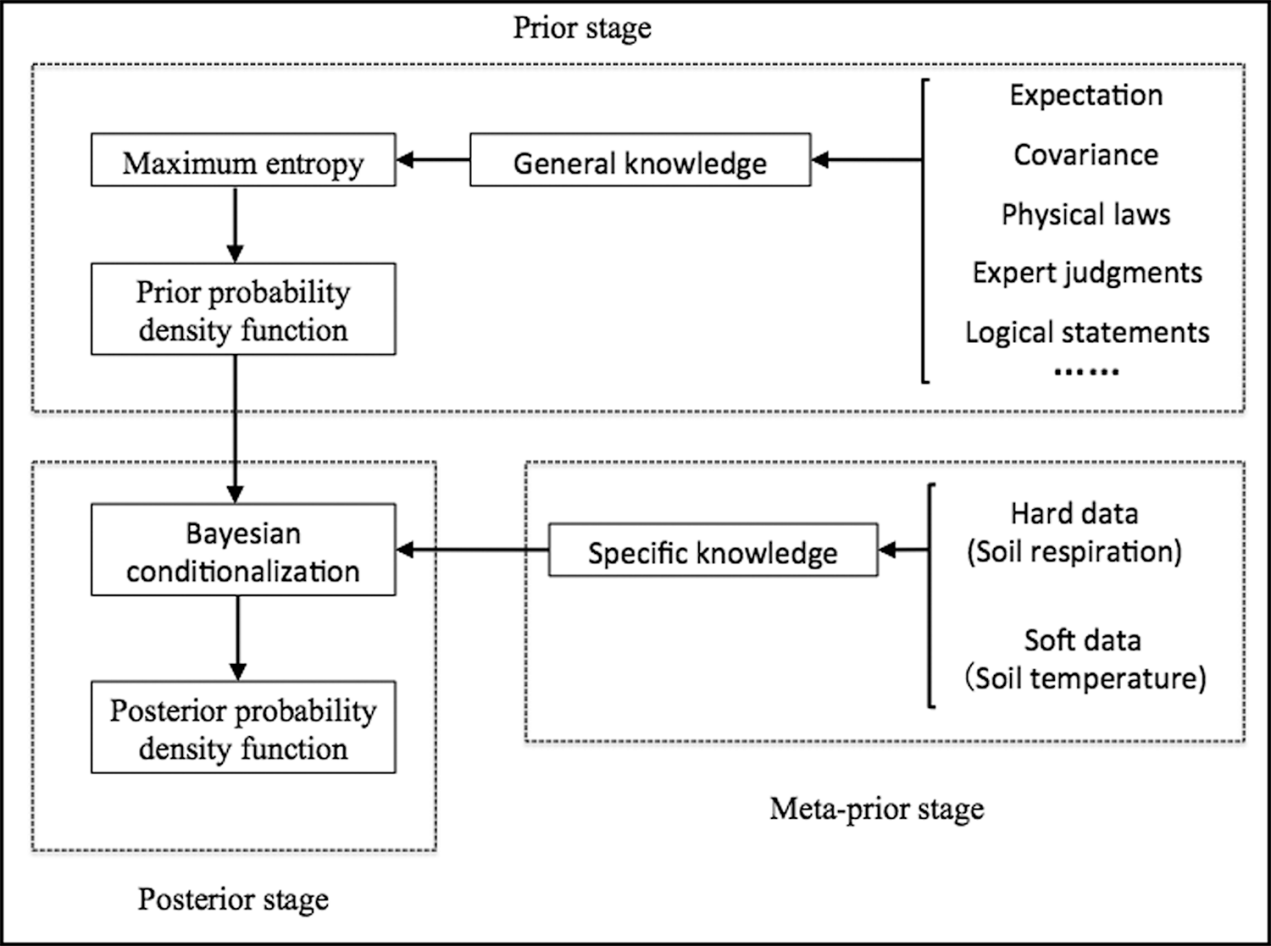
Flowchart of the BME Process.

The BME process can be divided into three stages: prior, meta-prior, and posterior (Fig 2). The prior stage mainly uses general knowledge *G*, to calculate the prior joint probability density function *f*_*G*_(*χ*_*map*_) using the Shannon information measure. *χ*_*map*_ is composed using hard data *χ*_*hard*_, soft data *χ*_*soft*_, and unknown values at estimation point *χ*_*k*_ (the point to be estimated). The process involved in obtaining the expected information is shown as Eq 9, where *g*_*α*_(*χ*_*map*_) contains the general statistical information about *χ*_*map*_, such as the mean and covariance. The Shannon information measure is used to maximize the entropy under the relevant constraints of *g*_*α*_(*χ*_*map*_). Eq 10 shows the function of the Lagrange multipliers method (LMM) for maximizing the expected information by introducing the Lagrange multiplier *μ*_*α*_ and the expectation of *g*_*α*_(*χ*_*map*_).

$$\bar{Info[\chi_{map}]}=-\int \;d\chi_{map}f_{G}(\chi_{map})log\;f_{G}(\chi_{map})$$ (9)

$$M[f_{G}(\chi_{map})]=-\int \;d\chi_{map}f_{G}(\chi_{map})log\;f_{G}(\chi_{map})-\sum_{\alpha }^{N}\mu_{\alpha }\{\int \;g_{\alpha }f_{G}(\chi_{map})d\chi_{map}-E[g_{\alpha }(\chi_{map})]\}$$ (10)

At the meta-prior stage, specific knowledge will be added into the calculation, including the measured hard data *χ*_*hard*_ and various forms of soft data *χ*_*soft*_. The soft data set *f*_*S*_(*χ*_*soft*_) denoted probability in this paper. At the posterior stage, using Bayes’ theorem, the posterior probability density function *f*_*K*_(*x*_*k*_|*χ*_*data*_) of the estimation points is calculated, taking into account the conditions of the specific knowledge, shown as Eqs 11 and 12:

$$f_{K}(x_{k}|\chi_{data})=A^{-1}\int \;d\chi_{soft}f_{S}(\chi_{soft})f_{G}(\chi_{map})$$ (11)

$$A=\int \;d\chi_{soft}f_{S}(\chi_{soft})f_{G}(\chi_{data})$$ (12)

After computing the posterior probability density function ***f***_***K***_(***x***_***k***_**|*χ***_***data***_), we can obtain the attribute values at the estimation points by way of the maximum posterior probability or maximum expectation, shown as Eqs 13 and 14:

$$x_{k}^{*}=\int \;x_{k}f_{K}(x_{k}|\chi_{data})dx_{k}$$ (13)

$$x_{k}^{*}=max(f_{K}(x_{k}|\chi_{data}))$$ (14)

where $x_{k}^{*}$ denotes the estimated values of ***x***_***k***_. We choose Eq 9 as the final estimation method in this paper.

#### Methods of validation

In order to evaluate the performance of the three methods (OK, Co-OK, and BME), about 10% of the collected data (which were selected to avoid continuous sampling points or points located on the outside, namely, a total of five points) were employed as data for cross-validation. The three statistical indicators of the root mean squared error (RMSE), correlation coefficient (CR), and average deviation (mean bias) were used to quantify the accuracy of the estimation results, as shown in Eqs 15, 16 and 17:

$$\mathrm{RMSE}=\sqrt{\sum_{k=1}^{n}\frac{{\left(x_{k}^{*}-x_{k}\right)}^{2}}{n}}$$ (15)

$$\mathrm{CR}=\frac{\sum_{k=1}^{n}(x_{k}^{*}-\bar{x^{*}})(x_{k}-\bar{x})}{\sqrt{\sum_{k=1}^{n}{\left(x_{k}^{*}-\bar{x^{*}}\right)}^{2}}\sqrt{\sum_{k=1}^{n}{\left(x_{k}-\bar{x}\right)}^{2}}}$$ (16)

$$\mathrm{Bias}=\frac{\sum_{k=1}^{n}(x_{k}^{*}-x_{k})}{n}$$ (17)

We applied GS+ 9.0 spatial analysis software to calculate the semivariance and for the OK and Co-OK methods[37]. The BMElib library (BMEGUI3.0 software) was used for the BME method[31], and Matlab 8.4 was employed for basic processing and mapping.

## Results

### Comparison of results of the OK, Co-OK, and BME methods

**Fig 3:**
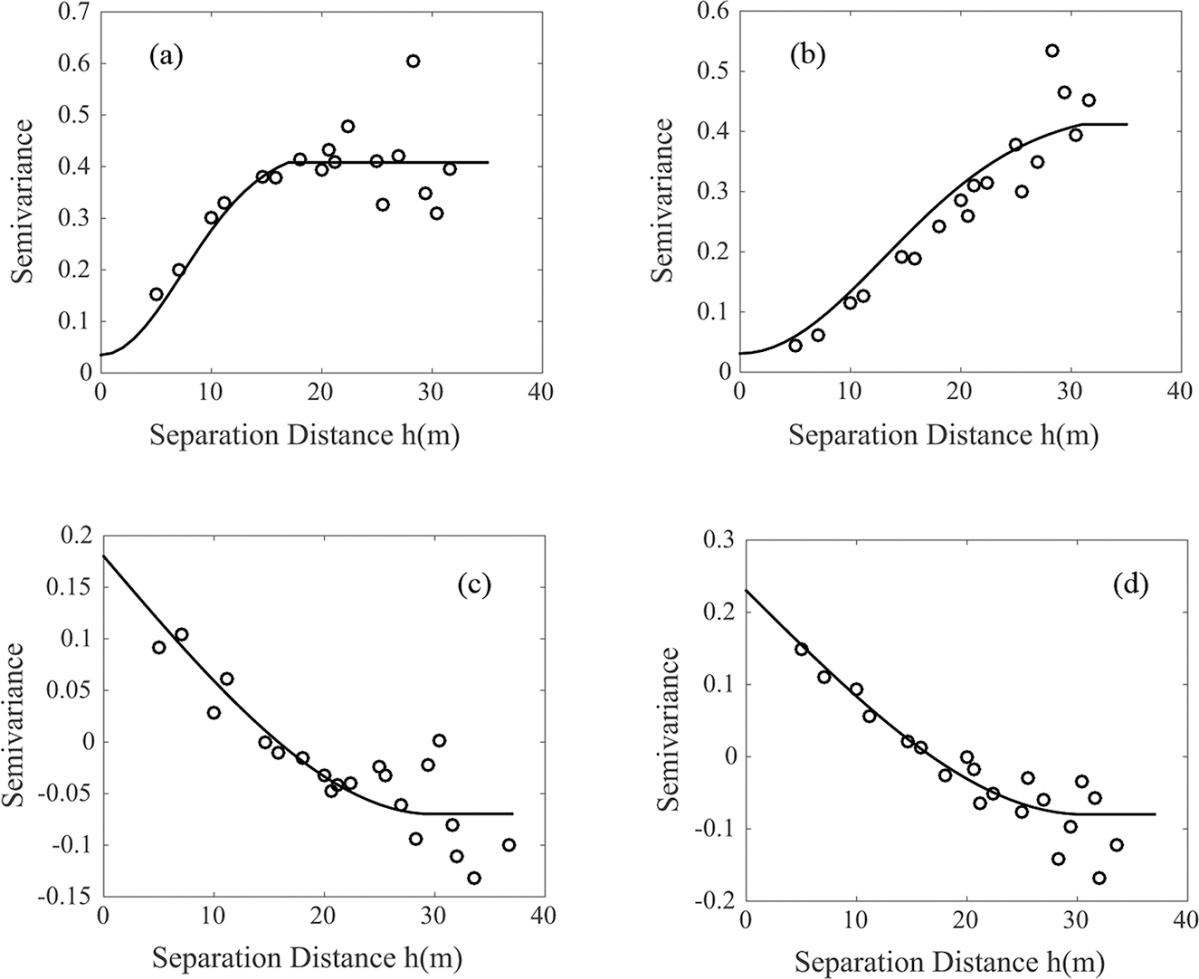
Auto- and cross-variograms fitted to the soil respiration and temperature (a) and (b) respectively are shown the variograms of soil respiration on Day1 and Day2. (c) and (d) respectively are shown the cross-variograms of soil respiration and soil temperature on Day1 and Day2.

the mean values of soil respiration in the study area was 3.476 and 5.81 μmg/m^2^s with the varition range of 2.698 and 3.019 μmg/m^2^s respectively, and the values of the coefficient of variation were observed 17.332 and 8.805 respectively, which proved that it was significant to consider the spatial variability of soil respiration in the study area. As shown in the Table 4, the parameters of the variogram models for the auto-variograms of soil respiration and the cross-variograms of the soil respiration and temperature in the study area during the abservation periods. There was some different in the range values of soil respiration in the observing days but the similar range of the cross-varigram of soil respiration and temperature, shown in Fig 3,and the soil respiration presented strong spatial dependence with the *C*_0_/(*C*_0_ + *C*) ratio<0.25.

**Table 4:** Models and parameters of the auto-variograms and cross-variograms fitted to the soil repiration and temperature.

ID	Variant	Models	C_0_	|C0+C|	C_0_/| C0+C|	A(m)	*R*^*2*^
**Day 1**	FCO_2_	Gaussian	0.035	0.436	0.080	18.05	0.706
**Day 1**	FCO_2_×Ts	Spherical	0.181	0.434	0.417	29.88	1.761
**Day 2**	FCO_2_	Gaussian	0.031	0.442	0.070	32.11	0.915
**Day 2**	FCO_2_×Ts	Spherical	0.232	0.542	0.428	30.59	0.887

Note: FCO_2_ is the CO_2_ Flux and the Ts is the soil temperature

**Fig 4:**
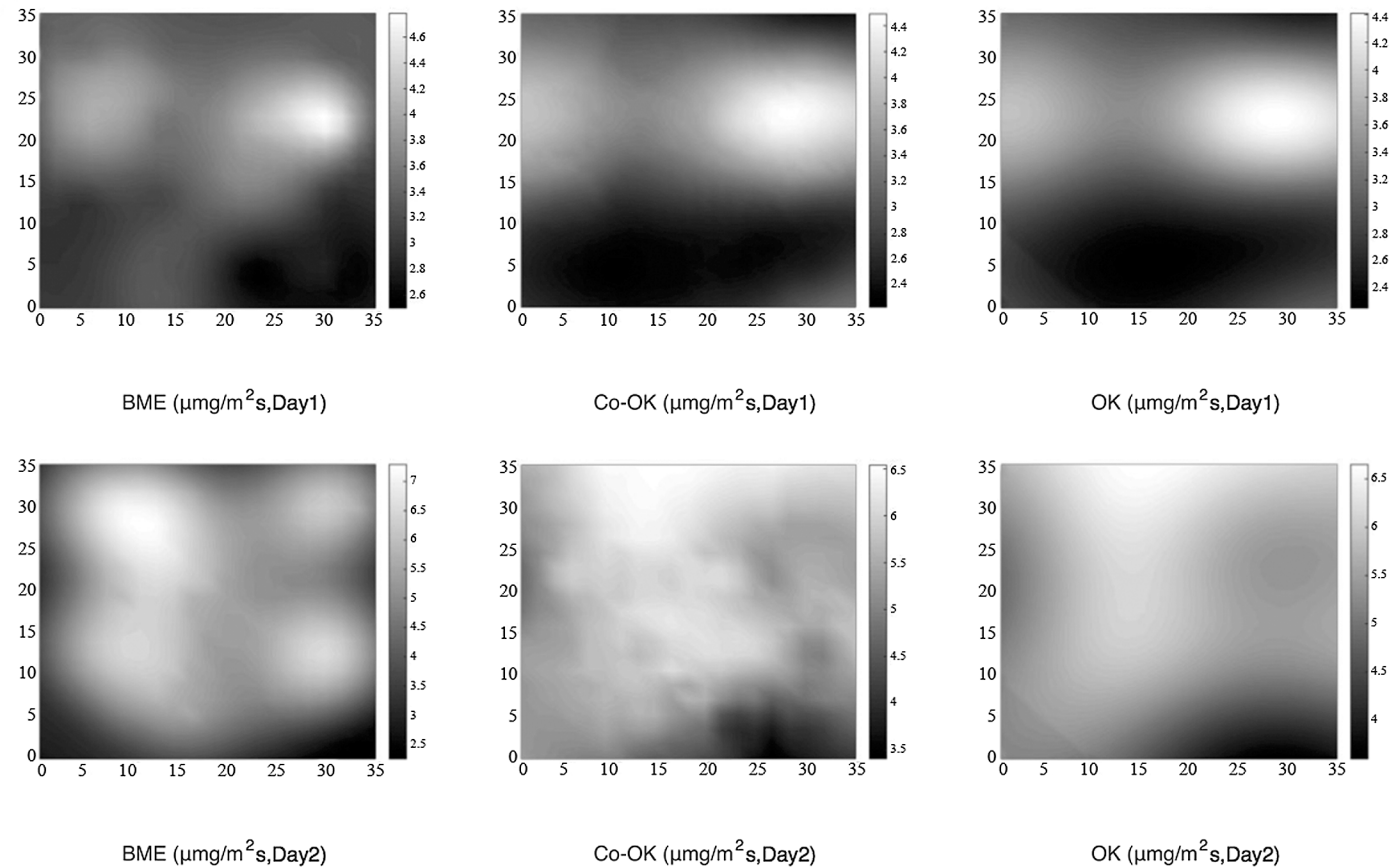
Spatial Distribution of Soil Respiration Using the BME, Co-OK, and OK Methods.

The spatial distribution of soil respiration in the study area was estimated using the three methods previously described. Fig 4 shows results of the mapping method for the samples collected over Day 1 and Day 2. In general, the three methods reflect the variations in soil respiration and its range in the study area, but certain difference in the local. The range of the spatial estimates, however, shows some differences among the three methods (Table 5). According to the BME method, the range on Day 1 and Day 2 was 3.543 and 5.038, respectively, which exceeded the ranges obtained using the Co-OK method (2.220 and 3.130, respectively) and the OK method (2.170 and 3.040, respectively). In fact, the results from the BME method showed an even greater range for both days than the corresponding values for the measured data (2.698 and 3.014, respectively). The estimations obtained using the Co-OK and OK methods fall in the same range as the measured values. Note that the values estimated using the Co-OK method are slightly larger than those obtained using the OK method. At the same time, the standard deviation of estimated value using the three methods also follow that the range of BME method is greater than Co-OK method than the OK method. In probability terms, it’s possible for the range of estimated value of soil respiration beyond the sampling data using only sampling data to estimate the value of soil respiration in the entire study area, so the BME method integrating more information and presenting the spatial variation of soil respiration in a probabilistic manner is more in line with real-world changes. Table 6 presents the validation results for the three methods. CR for Day 1 and Day 2 using the BME method (0.793 and 0.697, respectively) was significantly higher than the corresponding values obtained using the Co-OK and OK methods. The correlation between the estimation results from the BME method and the actual measurements is higher. Moreover, the RMSEs and absolute values of bias for both Day 1 and Day 2 were the lowest for the BME method, indicating that it can provide estimates of soil respiration in the study area with higher precision than the traditional Co-OK and OK methods.

**Table 5:** Summary of Soil Respiration Data.

ID	Data	Max(μmg/m^2^s)	Min(μmg/m^2^s)	Range(μmg/m^2^s)	Mean(μmg/m^2^s)	SD(μmg/m^2^s)
**Day 1**	Measured	4.939	2.242	2.698	3.476	0.602
**Day 1**	BME	4.939	1.396	3.543	3.093	0.579
**Day 1**	OK	4.490	2.320	2.170	3.133	0.422
**Day 1**	Co-OK	4.460	2.240	2.220	3.120	0.496
**Day 2**	Measured	6.649	3.635	3.014	5.810	0.512
**Day 2**	BME	7.290	2.252	5.038	5.072	1.094
**Day 2**	OK	6.650	3.610	3.040	5.530	0.616
**Day 2**	Co-OK	6.540	3.410	3.130	5.537	0.620

**Table 6:** Summary of Validation Results for the BME, Co-OK, and OK Methods.

ID	Method	RMSE	Bias	CR
**Day 1**	BME	0.727	-0.035	0.793
**Day 1**	Co-OK	0.911	-0.309	0.377
**Day 1**	OK	0.979	-0.296	0.078
**Day 2**	BME	0.409	-0.349	0.697
**Day 2**	Co-OK	0.790	0.707	0.632
**Day 2**	OK	2.042	-1.99	0.455

Note: RMSE, Bias, and CR refer to root mean square error, average deviation (mean bias), and correlation coefficient

### Effect of soil temperature as auxiliary information on the spatial estimation of soil respiration

In this study, we used only soil respiration samples for the OK method, while soil temperature data provided additional inputs to the BME and Co-OK methods. Table 6 shows that the RMSEs (using soil temperature data) obtained using the BME and Co-OK methods are 0.727 and 0.911, respectively, on Day 1, and 0.409 and 0.790, respectively, on Day 2, indicating that the inclusion of soil temperature as auxiliary information in the BME and Co-OK methods improves their overall performance compared to the OK method (its corresponding RMSE values being 0.979 and 2.042, respectively). The verification of the existing correlation indicates that the BME and Co-OK methods significantly outperform the OK method; thus, soil temperature data can effectively improve the reliability of the spatial pattern of soil respiration. However, the results for the deviation of validation (bias; Table 6) indicate only a small difference in results between the Co-OK and OK methods (-0.309 and -0.296, respectively, on Day 1), while the value for the BME method is -0.035, which is significantly better than those obtained using the other two methods. Thus, the auxiliary effect of soil temperature on the estimates would vary depending on the manner of its utilization.

**Fig 5:**
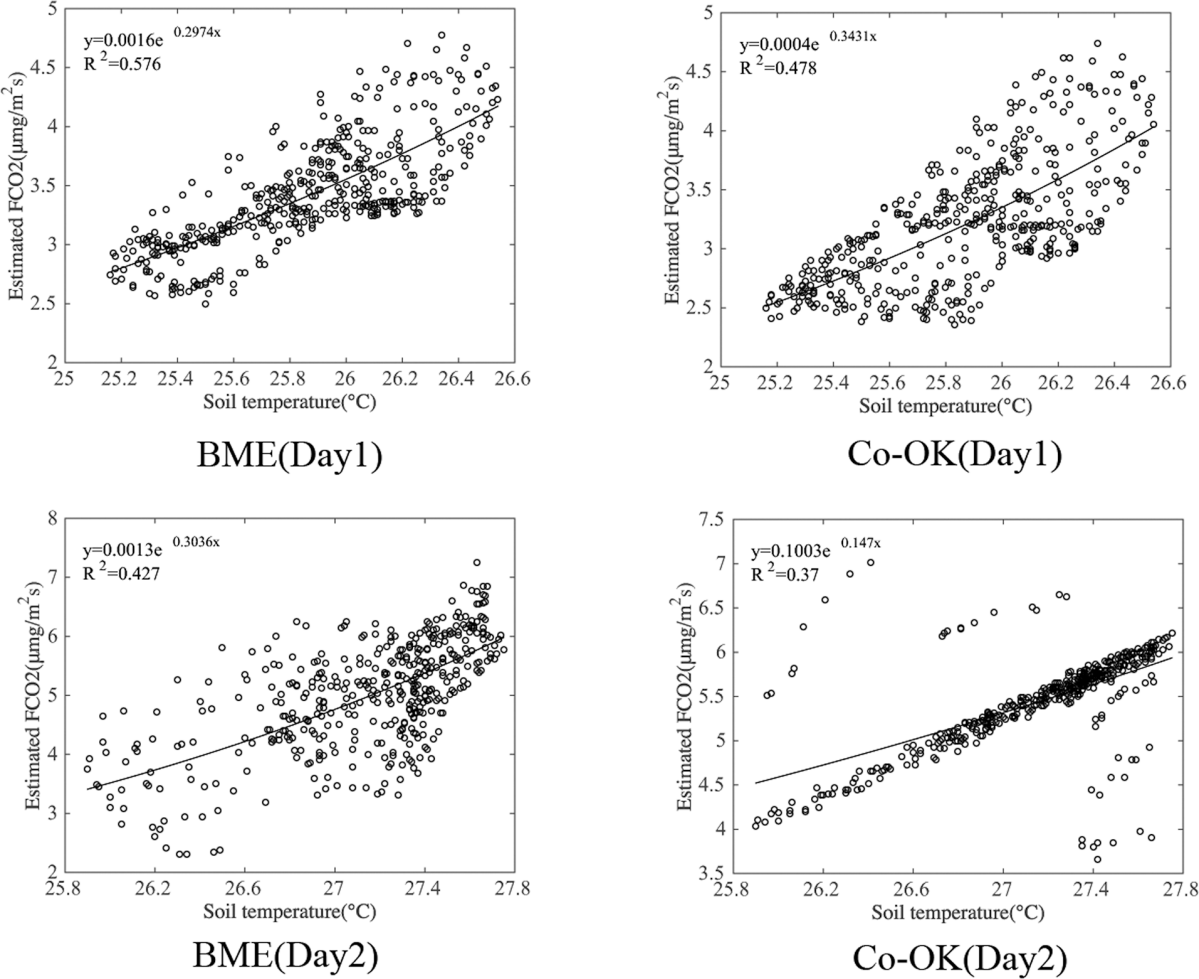
Fitting Relationship between Estimated Soil CO_2_ Efflux and Soil Temperature. Note: The total number of estimated points for each sampling strategy is 1225, and the analysis of correlation is calculated using Eq 12.

It is notable that the estimations obtained using the BME and Co-OK methods with soil temperature data are different. The results of the index regression model for both days of the measured data show that soil temperature can explain 60.6% and 57.1% of soil respiration respectively, as shown in Table 3. After estimating the spatial variation of soil respiration in the study area using the BME and Co-OK methods, the generated soil respiration estimates are exponentially related to the soil temperature values, see Fig 5. According to the results, in the BME method, soil temperature can explain 57.6% and 47.8% of soil respiration on Day 1 and Day 2, respectively. For the Co-OK method, the corresponding values are 47.8% and 37%, using the same soil temperature as auxiliary information. Thus, the utilization degree and degree of influence of the auxiliary information differ between the BME and Co-OK methods. Thus, Table 5 clearly indicates that, statistically, the BME method outperforms the Co-OK method in terms of estimating the spatial variation in soil respiration when soil temperature serves as auxiliary data.

### Effect of soil temperature as auxiliary information on soil respiration sampling points

**Fig 6:**
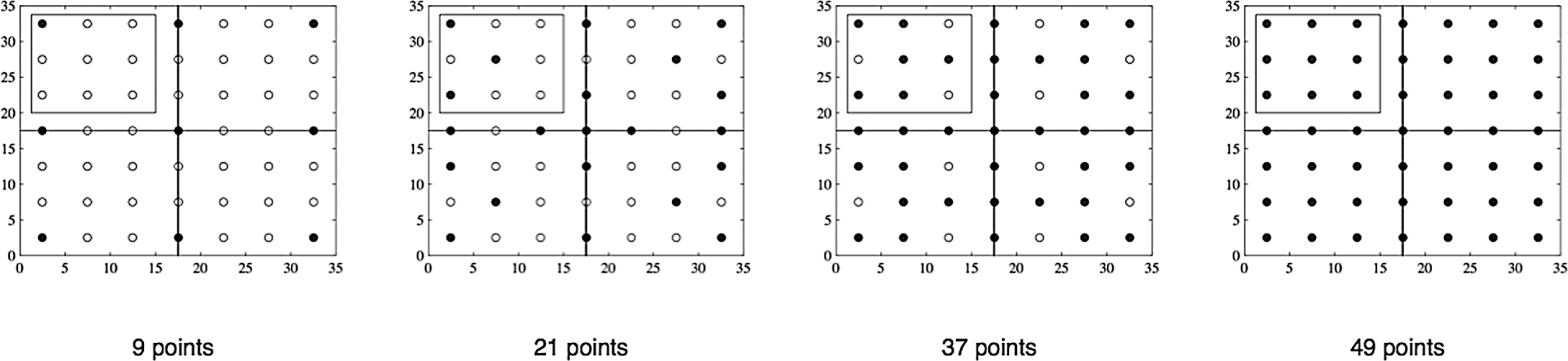
Distributions of 9, 21, 37, and 49 Measured Points. The solid circles depict the sampled points, and the clear circles, points where samples were not taken.

**Fig 7:**
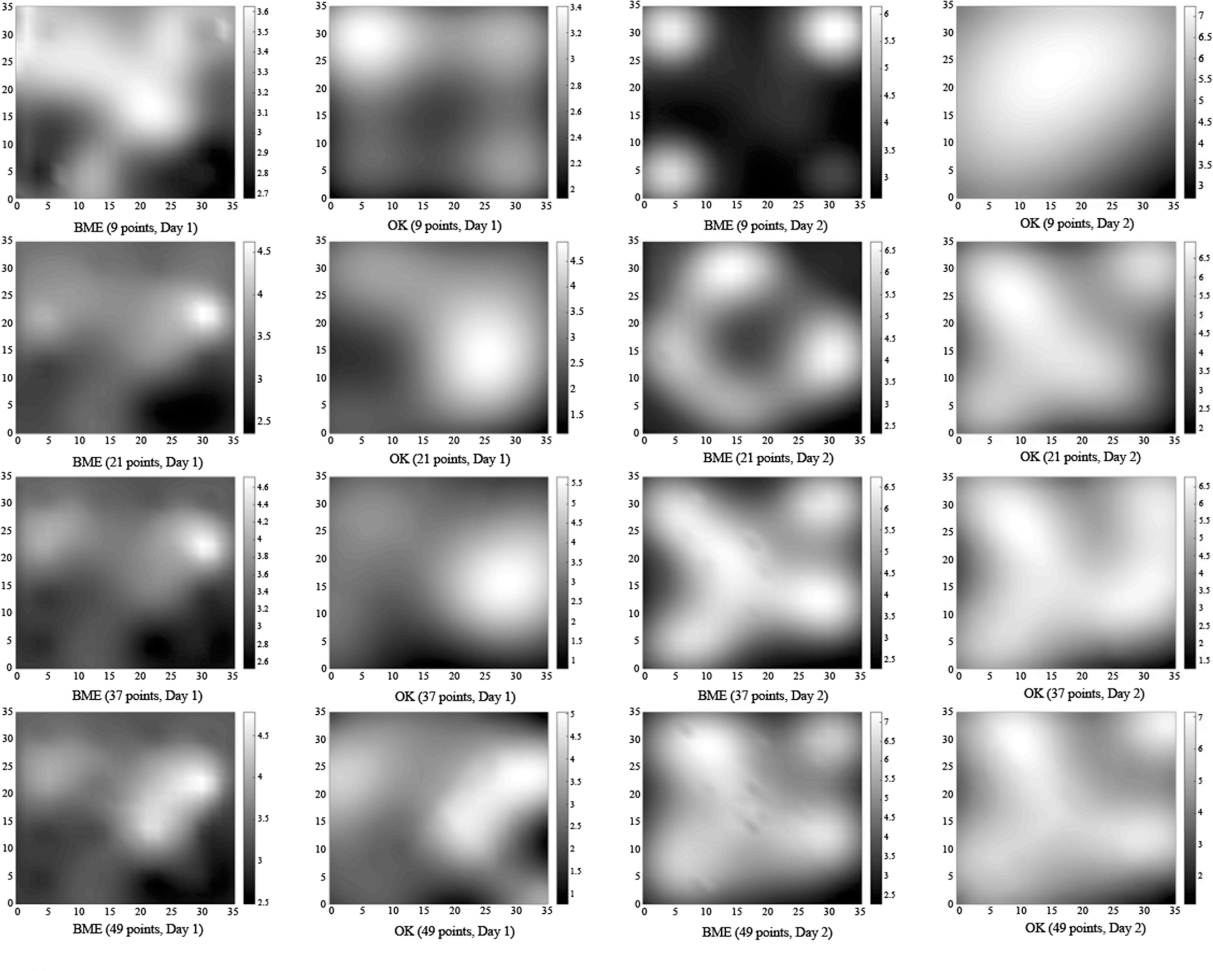
Spatial Distribution of Soil CO_2_ Efflux Using the BME and OK Methods When the Number of Measured Points is 9, 21, 37, and 49 (μmg/m^2^s).

**Fig 8:**
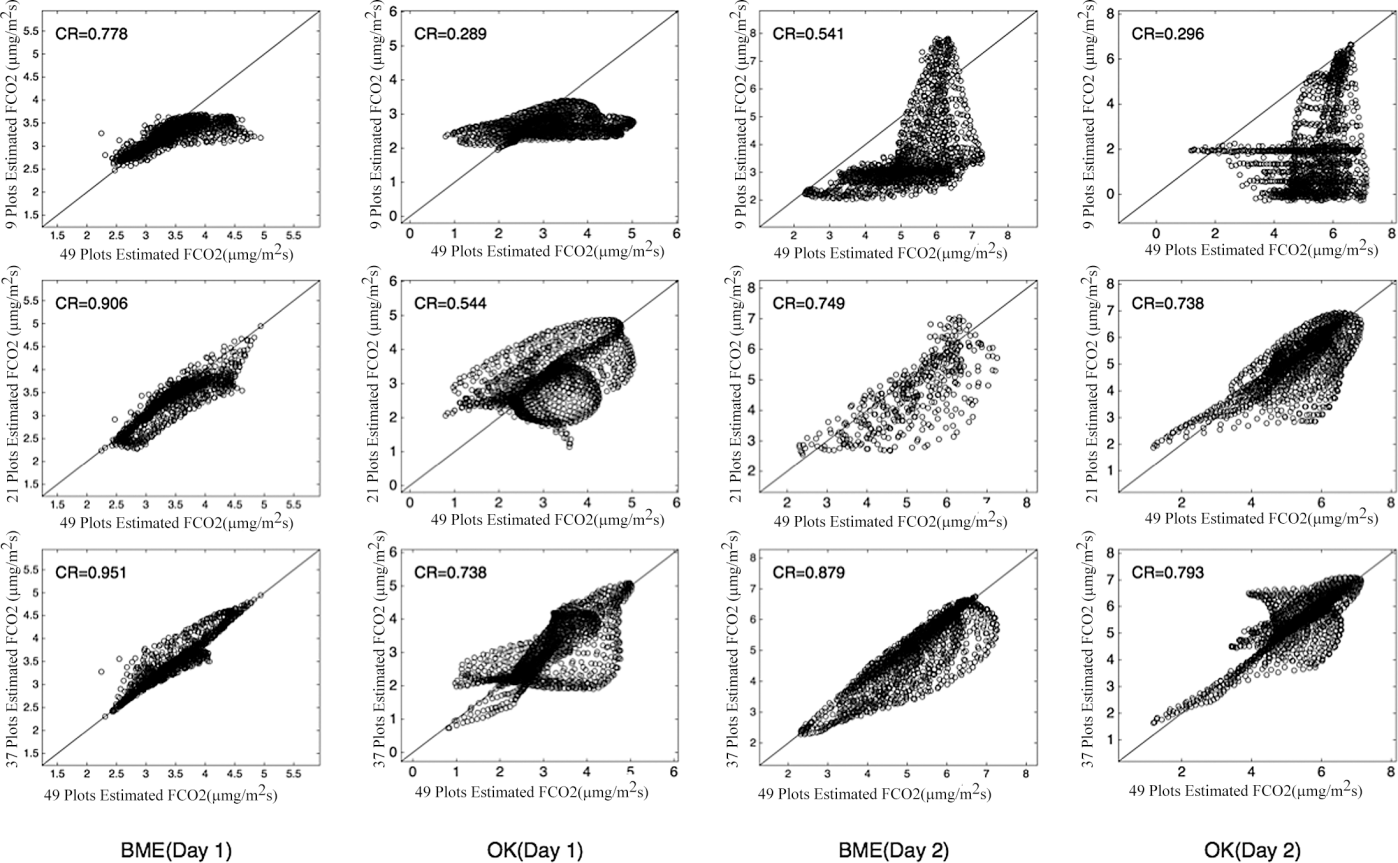
Correlation Between Estimated Soil CO_2_ Effluxes When the Number of Sampled Points is 9, 21, 37, and 49, Using the BME and OK Methods. Note: the Estimated Points is 1225 at Each Sampling Strategy, and the Analysis of Correlation Using Eq 12.

According to the analysis presented above, soil temperature can serve as auxiliary information to improve the estimation accuracy of the spatial distribution of soil respiration. In the field, soil temperature data can be obtained by setting up a wide network of wireless sensor networks, but these data are cumbersome to measure over multiple points over a large area and over a long period. However, the collection of high-density soil temperature data to help estimate soil respiration has certain practical significance. Accordingly, we reduced the number of sampling points for soil respiration in our study, and as noted in previous sections, using soil temperature data, we then applied the BME method to estimate the spatial distribution of soil respiration in the study area. The results were compared with estimates from the OK method, which could not use the temperature data. In order to ensure an even distribution of measuring points throughout the study area, we chose the measuring points across four quadrants (Fig 6), including points on the horizontal and vertical lines (axes) dividing the quadrants. The number of measuring points was increased from 1 to 3, from 3 to 6, and from 6 to 9 in each quadrant and by 1 on each axis, ultimately resulting in the 9, 21, 37, and 49 measuring points scheme adopted by this study. Fig 7 shows the spatial distribution of soil respiration estimated by the BME and OK methods when the number of measuring points was 9, 21, 37, and 49. The BME results continue to show more detailed information even when the number of sampling points are reduced. Conversely, the results from the OK method, which cannot account for temperature data, depend strongly on the number of measuring points, and thus, it is difficult to obtain detailed information with a reduced number of measuring points. Table 7 shows the validation results in terms of the RMSE and CR when the number of sampling points is 9, 21, and 37. For Day 1, the RMSEs for the BME method are 0.972, 0.838, and 0.673, respectively, while those for the OK method (without using soil temperature as auxiliary information) are 2.759, 1.246, and 1.146, respectively. This proves that the BME method using soil temperature as auxiliary information provides more accurate results, which are not significantly affected by the reduced number of soil respiration samples (that are difficult to obtain). The values of CR using the BME method with soil temperature as auxiliary information are 0.778, 0.906, and 0.951 when the number of sampling points is 9, 21, and 37, respectively. Again, the results of the BME method are preferable to those of the OK method (the corresponding values being 0.289, 0.544, and 0.738, respectively). Moreover, the differences in CR using the BME method for the three above-mentioned sampling strategies are smaller (Fig 8). We see similar patterns for Day 2 also. Furthermore, when the number of sampling points is 9, the RMSEs using the BME method are 0.972 and 1.193 for Day 1 and Day 2, which continue to be superior to the RMSEs obtained using the OK method when the number of sampling points is 37 (1.146 and 1.539, respectively). This means that the accuracy of the BME method, coupled with soil temperature as auxiliary information, does not suffer when the number of soil samples is reduced, and the effect of reducing the number of sampling points on the estimation results of the study area as a whole is small.

**Table 7:** Validation Results for Number of Sampling Points.

ID	Validation method	Interpolation method	9 points	21 points	37 points
**Day 1**	RMSE	BME	0.972	0.838	0.673
**Day 1**	RMSE	OK	2.759	1.246	1.146
**Day 1**	CR	BME	0.778	0.906	0.951
**Day 1**	CR	OK	0.289	0.544	0.738
**Day 2**	RMSE	BME	1.193	1.003	0.537
**Day 2**	RMSE	OK	2.216	1.646	1.539
**Day 2**	CR	BME	0.541	0.749	0.879
**Day 2**	CR	OK	0.296	0.738	0.793

## Discussion

### Accuracy of soil respiration spatial interpolation based on Bayesian Maximum Entropy is high

Christakos proposed the BME method in the early 1990s demonstrated both its theoretical and practical significance. Christakos also demonstrated that Kriging was a particular situation of BME under the limited assumption of transcendental information and effective data [32–36, 38]. By making the best use of auxiliary information, the BME method could effectively deal with real-world physical situations characterized by relatively high uncertainty. The BME method has been applied to the area of edaphology [39–41] and the results have demonstrated less error and higher accuracy compared with traditional interpolation. Spatial estimation values of higher accuracy and more detailed local differences have been achieved in its application to environmental science [42–47]. The BME method fittingly integrates into the soft data’s logarithm spatial analysis and improves the classification of results in data mining [48–50]. The BME method has also obtained better effects than traditional methods in applications to the ecological field [24, 51, 52].Traditional geostatistics mainly relies on sampled data measurements to obtain estimates for the whole study area without bias. The BME method, however, blends more soft data and expert knowledge with the sampled data, making full use of this information to enhance the estimation accuracy of the study object. For example, the spatial distribution of soil respiration can be studied using auxiliary information from the existing literature; Teixeira et al. [6] used soil bulk density as auxiliary information to improve estimates of spatial distribution of soil C density in sugarcane fields. This article considers the exponential relationship between soil temperature and C flux, by applying the BME approach to soil respiration and using soil temperature data as auxiliary information. BME thus effectively improves the accuracy of soil respiration spatial interpolation.

### Bayesian maximum entropy method coupled with soil temperature data (as soft data) can reduce the number of sampling points

Currently, the most widely used instruments in soil respiration monitoring are Li-8100 and Li-6400, which are so expensive that it is difficult to buy multiple instruments for simultaneous monitoring. The static air box method, although inexpensive, requires more manpower, and it is difficult to achieve simultaneous monitoring. Soil respiration typically exhibits strong spatial heterogeneity [19, 53–56]. Therefore, an insufficient number of monitoring points cannot characterize soil respiration for an entire area. Soil temperature, however, is relatively easy to measure, and these measurements can compensate for the lack of adequate monitoring points. Thus, devising a method that requires fewer monitoring points to achieve more comprehensive measurements of soil respiration is of great significance.

The biggest advantage of the BME method is that it can integrate soft data (prior knowledge, expertise, etc.) into hard data (measured data). Many scholars [32, 57–59] have concluded that, compared to conventional kriging, the BME method offers the advantages of higher accuracy and less error. Regarding the use of auxiliary information, the Co-OK method only uses weights to simply increase the influence of auxiliary information. The BME method, in contrast, is based on information entropy theory and using the LMM, it integrates the auxiliary information into the estimation of the natural attributes of interest. Then, using the Bayesian approach, it calculates the final estimate, thus making the whole process more systematic. During the integration of the auxiliary information, the BME method combines the measured data (the hard data ***X***_***hard***_ = {***X***_**1**_,***X***_**2**_⋯***X***_***h***_}) and the auxiliary information (soft data ***X***_***soft***_ = {***X***_***h*+1**_,***X***_***h*+2**_⋯***X***_***h*+*s***_}) into available information sets ***X***_***data***_ = {***X***_***hard***_, ***X***_***soft***_}, namely, the elements are expanded from *h* to *q* = *h* + *s*. Generally, the available soft data are very large; in this study too, the extent of soil temperature data significantly exceeded the measured soil respiration data. Notably, the expandation in the amount of information increases the uncertainty of information. Using its base of information entropy and the ability of the Bayesian approach to deal with information uncertainty, the BME method effectively combines the auxiliary information and measured data in the same space estimation [25, 52, 60]. Christakos also inferred the sum of partial derivatives of product ***μ***_***q***_***g***_***q***_(***X***_***k***_, ***X***_***hard***_, ***X***_***soft***_) between Lagrange parameter ***μ***_***q***_ and statistical matrix equation ***g***_***q***_(***X***_***k***_, ***X***_***hard***_, ***X***_***soft***_) with respect to the estimation point ***X***_***k***_; in other words, the BME method entails the simultaneous inclusion of massive amounts of soft data and hard data into the calculation [38]. Similarly, in this study, high-density soil temperature data were integrated into the space estimates alongside soil respiration values. Meanwhile, in the absence of adequate amounts of hard (measured) data region, the integration of soft data compensates for this absence of hard data to some extent. Christakos et al. [32] also noted that the BME method uses less measured data to obtain better estimates than the simple kriging method, which uses the space of all measured data. In agreement with this result, this study showed that the BME method, when coupled with soil temperature data and measured data for only nine measurement points, had better effects than the OK method for 37 measurement points as the verification results showed in Table 6. Thus, the BME method, coupled with soil temperature as auxiliary information, can reduce the number of monitoring points in soil respiration studies by a considerable extent.

## Conclusions

This paper coupled the BME method with auxiliary information of soil temperature, to study the spatial distribution of soil respiration. The results indicated that the BME method is superior to the Co-OK and OK methods. Moreover, such application can reduce the number of soil respiration sampling points, thus indicating its significance in the area of soil respiration monitoring, the equipment for which is typically expensive enough to restrict researchers to sparse single-point monitoring.

This study also has some limitations. We chose soil temperature as auxiliary information. However, the spatial heterogeneity of soil respiration is affected by many other factors, such as soil C storage, soil bulk density, and root and microbial biomass. These factors can be taken into consideration in future studies of the performance of the BME method in interpolating soil respiration distributions while using data on multiple factors as auxiliary information.
